# Ranolazine-induced Elevation of Creatinine Kinase in the Absence of Statin Usage

**DOI:** 10.7759/cureus.2832

**Published:** 2018-06-18

**Authors:** Eric Dein, Rebecca Manno, Abrahim Syed, Homeyra Douglas, Duvuru Geetha, Homa Timlin

**Affiliations:** 1 Internal Medicine, Johns Hopkins Bayview Medical Center, Baltimore, USA; 2 Rheumatology, The Johns Hopkins University School of Medicine, Baltimore, USA; 3 Cardiology, University Hospital Aintree, Liverpool, GBR; 4 Medicine, The Johns Hopkins University School of Medicine, Baltimore, USA; 5 Medicine, The Johns Hopkins University School of Medicine, Essex, USA

**Keywords:** ranolazine, elevated ck

## Abstract

Ranolazine received Food and Drug Administration (FDA) approval in 2006 for the treatment of chronic angina. Ranolazine has previously been linked to the development of statin-induced myopathy, because it also inhibits CYP3A4, which increases serum statin levels. In the absence of concomitant statin therapy, elevated creatinine kinase (CK) and myalgias on ranolazine monotherapy has never been reported.

## Introduction

Ranolazine is prescribed for the treatment of chronic cardiac angina. When used in conjunction with concomitant statin therapy, it has been demonstrated to increase serum statin levels, and has been associated with rhabdomyolysis [1-3]. This is the first report of myalgias and elevated creatinine kinase reported without concomitant statin therapy.

## Case presentation

A 33-year-old female with sickle cell trait, anxiety, miscarriages (twice), late-onset Raynaud’s phenomenon, and fibromyalgia, initially presented with chest pain and elevated troponin level. Her local physician found a negative cardiac workup, and subsequently she was initiated on ranolazine for treatment of suspected coronary vasospasm.

She presented to a local hospital for evaluation of intermittent and increasing non-exertional chest pain and mild shortness of breath about four weeks after hysterectomy. On admission, she was noted to have an elevated troponin I level at 0.28 (normal <0.02), which downtrended to 0.26. She had a cardiac evaluation with a technetium-99m sestamibi stress test that revealed no evidence of ischemia. Computed tomography (CT) with contrast showed no evidence of pulmonary consolidation or pulmonary embolism. She was diagnosed with suspected coronary vasospasm. Her chest pain resolved on ranolazine 500 mg twice daily, however, within one week, she developed generalized myalgia. Follow-up labs revealed creatinine kinase (CK) levels to be 4551 U/L (range 26-308). Aldolase elevated at 32.4 U/L (range 3.3-10.3). Other pertinent labs included an elevated aspartate aminotransaminase of 101 U/L, normal alanine aminotransaminase, positive antinuclear antibody (ANA) (1:160, homogenous), and erythrocyte sedimentation rate (ESR) at 1 mm/hr. The ranolazine was held with the improvement of myalgia symptoms. Repeat lab testing two weeks later was notable for CK of 76 U/L. Evaluation by neuromuscular and rheumatologist confirmed undifferentiated connective tissue disease based on positive ANA, late-onset Raynaud’s, alopecia, and no signs to suggest an autoimmune inflammatory myositis.

Her serology was negative for anti-double-stranded DNA, anti-Jo-1, anti-Scl-70, anti-Smith, anti-SS-A, anti-SS-B, and anti-cyclic citrullinated peptide (CCP). Other workup included normal C3, C4, comprehensive metabolic panel (CMP), complete blood count (CBC), ESR, C-reactive protein (CRP), anti-cardiolipin panel, anti-B2glycoprotein, dilute Russell viper venom time (dRVVT), and comprehensive Oklahoma Medical Research Foundation (OMRF) myositis antibody panel. She was evaluated by a neuromuscular team. Cardiac and lower extremity magnetic resonance imaging (MRI) showed no evidence of inflammation. Electromyography (EMG) with nerve conduction study (NCS) and left thigh muscle biopsy also did not reveal evidence of inflammatory myopathy.

At last follow-up, the patient had no recurrence of myalgias 10 months after discontinuing ranolazine. CK and aldolase have remained within normal limits at 82-138 and 5.8-7.4 (aldolase range 3.3-10.3), respectively.

## Discussion

Ranolazine is a commonly used anti-anginal that received Food and Drug Administration (FDA) approval in 2006 for the treatment of chronic angina. Ranolazine works through the inhibition of late sodium current in cardiac myocytes, which increases the efficiency of oxygen use [4]. In the Ranolazine Open Label Experience (ROLE) study [5], Koren et al. reported the safety and tolerability data from 746 patients on ranolazine therapy with mean follow-up of 2.82 years. They reported that 571 patients (76.7%) remained on ranolazine therapy and 72 patients (9.7%) reported discontinuing for adverse events. The most common adverse events that were reported were angina pectoris (14.9%), dizziness (11.8%), constipation (10.9%), peripheral edema (8.3%), and fatigue (7.05%). Myalgias and rhabdomyolysis were not listed as common adverse events. In a large efficacy study by Wilson et al., they reported on 3,565 patients on ranolazine with a median follow-up of 350 days [6]. They reported a discontinuation rate of 8.1% from adverse effects, most commonly due to dizziness (12.4%), nausea (9.7%), and constipation (8.5%). Like Koren et al., they did not report any discontinuations secondary to rhabdomyolysis or myalgias.

Elevated CK and rhabdomyolysis has been described in four case reports in patients on ranolazine therapy [1-4]. In each prior case, the patients were also on statin therapy. The proposed mechanism of the adverse effect was an increase in serum concentration of statin secondary to drug interaction. Ranolazine is metabolized by CYP3A4 and CYP2D6 and weakly inhibits CYP3A4, which is responsible for the metabolism of simvastatin and atorvastatin [7].

We report the case of a patient who developed myalgias and an elevated CK to 4551 U/L secondary to ranolazine. This corrected with the discontinuation of the medication. This is the first reported case of an elevated CK from ranolazine without concomitant statin therapy. This case suggests an independent cause of creatinine kinase elevation in patients on ranolazine therapy, rather than potentiating statin-induced myopathy alone.

## Conclusions

Our presented case demonstrated CK elevation on a patient off of statin therapy, suggesting an alternative mechanism of ranolazine inducing myopathy.
